# Clinical Rationale for Procedural Steps When Performing Non‐Incisional Nail Surgery: A Scoping Review

**DOI:** 10.1002/jfa2.70191

**Published:** 2026-07-27

**Authors:** Anna Horn, Caroline Robinson, Luke Donnan

**Affiliations:** ^1^ School of Allied Health & Exercise Sport Sciences Charles Sturt University Thurgoona New South Wales Australia

**Keywords:** ingrown toenail, matrixectomy, nail pathology, nail surgery, onychocryptosis, podiatry

## Abstract

**Background:**

Non‐incisional nail surgery is a common surgical procedure performed by clinicians, however, there is considerable variation relating to decision‐making for procedure choice, surgical technique, and clinical rationale to inform practice. The aim of this scoping review is to map the extent and type of evidence relevant to surgical approach, decision‐making, and clinical rationale informing non‐incisional nail surgery in clinical practice.

**Methods:**

The inclusion criteria were full text papers, available in English that discussed non‐incisional nail surgery with or without matrixectomy and included procedural steps and evidence of a clinical rationale to inform practice. A systematic search was conducted of the following databases: Medline, Web of Science, Emcare, CINAHL, ProQuest (Health and Medical), ProQuest (Nursing and Allied Health) and Health Collection. The reference lists of review articles were screened for additional studies. Relevant data were extracted, charted, and grouped into categories. Compiled data were then transferred into summary tables as frequencies and percentages.

**Results:**

A total of 110 articles were included in this review. The articles were published between 1965 – 2025. The procedural steps and clinical rationale were grouped into themes, highlighting a considerable variation in practice relating to tourniquet use, matrixectomy method, concentration of caustic agent, application time and irrigation practices following cauterisation.

**Conclusions:**

This review has identified inconsistency in the documented practice of non‐incisional nail surgery based on published literature spanning a period of 37 years. Specifically, this surgical intervention appears to be informed by historical practice and there is little evidence of a common approach. The authors propose that clinical practice guidelines are needed to provide an evidence‐based approach to decision‐making in non‐incisional nail surgery. The development of clinical guidelines including a structured decision‐making framework, would support consistency in practice and facilitate evidence‐based decision‐making.

## 1 Introduction

An ingrown toenail (IGTN), or onychocryptosis, occurs when the border of a nail injures the adjacent skin and surrounding soft tissue, resulting in inflammation and a risk of infection [1]. Treatment options vary depending on the severity of the IGTN and range from conservative approaches to surgical interventions. Conservative approaches may include the use of guttering techniques, footwear education and proper nail trimming [2]. The most common indications for performing a nail surgery procedure include an acute IGTN, repeat episodes of IGTN, failed conservative treatments, and other chronic nail conditions such as onychogryphosis [3].

Surgical interventions for nail pathology can be broadly classified into incisional and non‐incisional procedures. Incisional nail surgery involves a surgical incision into soft tissue to allow mechanical excision of the affected nail plate, nail matrix and nail bed tissues. Additionally, incisional procedures may also include resection of the surrounding soft tissue to correct nail folds [2, 4]. In a non‐incisional nail surgery, either a section of nail or the total nail plate is removed, without incision of the nail bed or proximal nail fold. If a matrixectomy is indicated, the germinal nail matrix is destroyed using a chemical solution (phenol; sodium hydroxide) or a physical ablative technique (radiofrequency; electrosurgery) [4, 5].

The clinical decision to proceed with nail surgery, and selection of the appropriate procedure, is typically based on the presenting condition, prior treatment history and the clinician's expertise and experience [1, 6]. Any nail surgery procedure has both benefits and risks, emphasising the need for personalised treatment plans appropriate to the client's needs and presenting nail condition. The selection of a specific surgical technique is informed by the intended outcome of the surgery, the practitioner's favoured surgical approaches, and their experience [1, 6].

In a recent systematic review and meta‐analysis of randomised controlled trials on surgical treatments for IGTNs [7], the authors noted a lack of good quality evidence to guide clinical decision‐making. This may reflect the considerable variations in surgical practice including: type of local anaesthetic used to perform a digital block; use of a digital tourniquet; matrixectomy technique; duration of cauterant application; sharp debridement of adjacent tissue; and post‐matrixectomy irrigation [3].

A lack of clarity about decision‐making in the practice of non‐incisional nail surgery creates the potential for inappropriate procedure selection and sub‐optimal surgical outcomes, such as IGTN recurrence or post‐operative complications. This scoping review aims to illuminate and explore the breadth of published literature relevant to surgical approaches, decision‐making and clinical rationale for choices made in non‐incisional nail surgery.

## 2 Methods

This review was conducted in accordance with the Joanna Briggs Institute (JBI) Methodology for Scoping Reviews [8]. The protocol was registered on Open Science Framework on the 12th of May 2025 https://osf.io/7frjb.

A preliminary search of MEDLINE, the Cochrane Database of Systematic Reviews and JBI Evidence Synthesis was conducted and no current or underway systematic reviews or scoping reviews on the topic were identified. An initial limited search of MEDLINE (PubMed) and CINAHL (EBSCO) was undertaken in conjunction with a university librarian to identify relevant literature. The text words contained in the titles, abstracts and index terms of relevant articles were used to develop a full search strategy. The search strategy (Supporting Information S1: Appendix A) was adapted for each of the following databases: Medline, Web of Science, Emcare, CINAHL, ProQuest (Health and Medical), ProQuest (Nursing and Allied Health) and Health Collection on the 17th of March 2025.

The inclusion criteria prioritised papers that discussed non‐incisional nail surgery with or without matrixectomy, including procedural steps and proposed clinical rationale for their application. Relevant manuscripts were selected based on the predetermined research questions:

1. What are the procedural steps used by clinicians when performing non‐incisional nail surgery with or without matrixectomy?
2. What is the proposed clinical rationale for applying the procedural steps in non‐incisional nail surgery with or without matrixectomy?

This scoping review considered a broad range of studies and all non‐review type study designs were included. No time limitation for publication date was set, to allow inclusion of historical contributions and foundational principles. Only sources available in journal databases, published in English and available in full text, were included in the review.

All identified literature was collated and uploaded into the Covidence systematic review software [9]. Duplicates were automatically removed and missed duplicates were removed manually. In the first stage of the review process, all titles and abstracts was assessed against the inclusion criteria by two independent reviewers. The full text of articles included by the reviewers during the title and abstract screening process was imported into Covidence. In the second stage of the review process, two independent reviewers assessed each of the full text articles against the inclusion criteria.

The reference lists of excluded review articles were screened for additional studies. Any additional studies were included for full text review.

**FIGURE 1:**
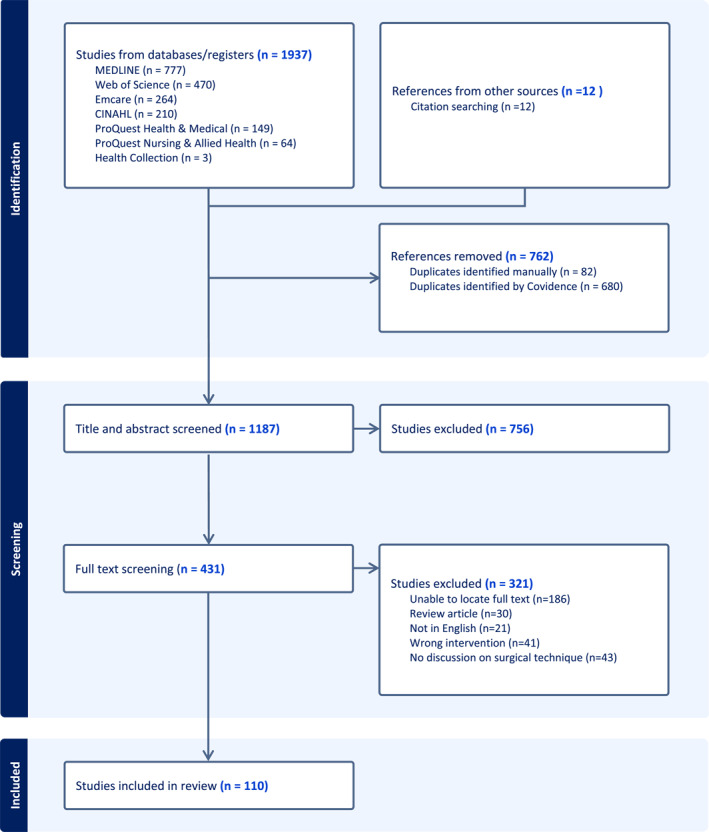
PRISMA‐ScR diagram for search strategy. *n*, number.

The results of the search and the study inclusion process are reported in full and in accordance with the Preferred Reporting Items for Systematic Reviews and Meta‐analyses extension for scoping review (PRISMA‐ScR) [10] and presented in a PRISMA diagram (Figure 1).

### 2.1 Data Extraction

A data extraction spreadsheet (Microsoft Excel) was designed to facilitate data collection by either two or three independent reviewers (AH, CR, LD) (Supporting Information S2: Appendix B and Supporting Information S3: Appendix C). The data extraction tool was independently piloted by two reviewers (AH & LD), and subsequently adjusted to improve functionality. All three reviewers met to confirm the extraction process before commencing. Data extraction was based on pre‐determined categories and included: author, publication year, country, study title, study design/methodology, intervention type, comparison type, details of procedural steps, and rationale to support each of the procedural steps. Clinical rationale refers to subjective information reported by authors of individual articles. Each full text source was evaluated by a pair of reviewers and any disagreements were resolved by the third reviewer.

## 3 Results

### 3.1 Data Analysis

All extracted data were charted according to the surgical procedural steps, with the corresponding rationales grouped into categories. Charted data were transferred into summary tables as frequencies and percentages (Tables 1, 2, 3). If specific surgical steps or rationale were not explicitly reported, these were reported as absent.

**TABLE 1:** Summary and frequency table for non‐incisional nail surgery procedural steps and clinical rationale.

Surgical process (product, instrument or item)	Frequency (*n*)
Part A: Toe preparation	56
Povidone‐iodine	29
Choice of solution: Chlorhexidine/alcohol/iodine	7
Chlorhexidine	6
70% alcohol	5
Sterile conditions/prepared in the usual way/draped	5
Standard foot scrub/disinfectant scrub	4
Clinical rationale for toe preparation	10
Disinfect toe (*n* = 6); clean toe (*n* = 2); soften nail (*n* = 1); isopropyl alcohol scrub and chlorhexidine is superior to povidone‐iodine solution (*n* = 1)	
Part B: Tourniquet application	46
Rubber band or latex strip	28
Tourniquet (sterile/digital/Esmarch, coloured ring or mini‐tourniquet)	16
Catheter	2
Clinical rationale for tourniquet application	76
Haemostasis/exsanguination (*n* = 46); blood neutralises or inactivates chemical cauterant (*n* = 6); blood dilutes agent (*n* = 3); facilitates optimum activity of chemical cauterant (*n* = 3); limiting application time/or only applied immediately before caustic agent (*n* = 3); blood counteracts the effect of chemical cauterant (*n* = 2); caution in people with diabetes or peripheral vascular disease (*n* = 2); type of tourniquet not used due to risk of failing to remove (flesh coloured glove tourniquet) (*n* = 1); restricts local anaesthesia to the toe so large volumes of local anaesthesia are unnecessary (*n* = 1)	
Clinical rationale for not applying a tourniquet	
Ischaemia concerns due to tissue compression (*n* = 6); tourniquet not required (with added adrenaline or due to short duration of nail removal procedure) (*n* = 3)	
Part C: Nail plate elevation	45
Nail elevator	33
Forceps/artery clip/haemostat	7
Nail clippers/scissors	2
Blacks file/probe	2
Mini‐osteotome	1
Clinical rationale for nail plate elevation	40
Release nail from nail bed and other soft tissue structures (eponychium/lateral nail fold) (*n* = 38); nail elevator is less traumatising (*n* = 2)	
Part D: Nail plate section	48
Nail splitter	25
Nail nippers/scissors/side cutter	19
Scalpel/blade (number.11)	2
Carbon dioxide (CO_2_) laser	1
Radiofrequency probe	1
Clinical rationale for nail plate section	52
Section the toenail from the free edge to the eponychium by creating a longitudinal split (*n* = 39); minimise trauma to the nail bed (*n* = 5); avoid cutting overlying skin/epidermal tissue (*n* = 4); provide access to the matrix horn (*n* = 3); 5 and 7 W power of CO_2_ laser was too low to cut the nail plate and 10 W was good enough power (*n* = 1)	
Part E: Beaver blade	10
Beaver blade/#62 blade, chisel blade	10
Clinical rationale for beaver blade	8
Cut the section of nail plate deep to the proximal nail fold (*n* = 7); jaws of the nail splitter are too large to fit beneath the proximal nail fold without tearing. Narrower chisel blades tend to lacerate the nail bed and matrix less (*n* = 1)	
Part F: Nail removal	57
Forceps/artery clip/haemostat/clamp	47
Instrument not noted but method described	7
Nail splitter	1
Needle holder	1
Blunt dissection	1
Clinical rationale for nail removal	24
Avulse/expose the germinal matrix or matrix horn (*n* = 10); removal process explained to minimise the chance of leaving a nail fragment or splinter (*n* = 6); ensure nail removal is lower than the basal matrix (*n* = 2); removal process to prevent spicule from tearing flesh of toe (*n* = 2); allows access to the nail matrix through proximal nail fold without need for any incision (*n* = 2); ensure nail is fully removed, as remnant nail will prevent ablation (*n* = 1); the width of nail removed only needs to be slightly great than is embedded into nail fold (*n* = 1)	
Part G: Removal of additional tissue	47
Scalpel or curette	38
Scissors/nail nippers	3
Chemical cautery	2
Electrocautery	1
CO_2_ laser	1
Radiosurgery	1
Haemostat	1
Clinical rationale for removal of additional tissue	63
Removal of granulation/hypertrophied tissue or granulomas (*n* = 39); to remove debris/remaining matrix (*n* = 12); to optimise visualisation of the nail matrix (*n* = 3); to verify no remaining nail spicules (*n* = 3); to remove excess nail fold to prevent reoccurrence of nail fold overgrowth (*n* = 3); to remove cauterised tissue (*n* = 1); to remove thin membranous layer of epithelium to allow outflow of serum and blood postoperatively to reduce pressure or haematoma development (*n* = 1) to expose all remaining matrix to cauterant (*n* = 1)	

*Note:* Frequency *n*: identifies the number of articles from the original sample (*n* = 110) that describe a procedural step (product, instrument, item) that is performed during a non‐incisional nail surgery. A single article may be counted in multiple categories if it addresses more than one product, instrument or item. Clinical rationale n: identifies the number of times a supporting rationale is presented for a procedural step (product, instrument, item) that is performed during a non‐incisional nail surgery. A single article may present multiple rationales.

Abbreviations: % = percent, *N* = number, W = watt.

**TABLE 2:** Summary and frequency table of matrixectomy techniques, clinical rationale and procedural considerations.

Part A: Chemical matrixectomy			
Phenol			
Concentration	*n*	Application time	*n*
100%	2	3 min	31
90%	3	1 min	17
89%	6	Range (30 sec–10 min)	10
88%	39	2 min	10
85%	1	1.5 min	7
80%	6	Not noted	5
70%	1	5 min	2
45%	1	4 min	2
80%–88%	4	30 sec	2
80%–100%	1	3 min plus 30 sec	1
88%–90%	3	45 sec	1
Concentration not reported	14	6 min	1
		1.5 min PNA; 2.5 min TNA	1
Clinical rationale for the use of phenol			31
Advantage offered by chemical properties (*n* = 14); cauterisation of nail matrix (*n* = 9); advantages over other surgical options (*n* = 6); reduced risk of recurrence (*n* = 2)			
Procedural considerations for phenol			51
Balancing application time with tissue destruction (*n* = 14); technique to minimise damage to surrounding tissues (*n* = 13); bloodless field required prior to phenol application (*n* = 9); potential risks associated with use of phenol (*n* = 5); choice of phenol related to patient selection (*n* = 3); selection based on presenting nail pathology (*n* = 3); visible tissue colour change with application of phenol (*n* = 2); concentration required for adequate cauterisation (*n* = 1); method of application of phenol (*n* = 1)			

Sodium hydroxide (NaOH)			
Concentration	*n*	Application time	*n*
10%	20	1 min	11
Not noted	1	2 min	2
		Various (blanching of tissues)	2
		30 sec	1
		4 min	1
		20–25 sec	1
		30 sec, 1 min or 2 min	1
Clinical rationale for the use of sodium hydroxide			8
Advantages when compared with phenol (*n* = 7); cauterisation of nail matrix (*n* = 1)			
Procedural considerations for sodium hydroxide			9
Suitable for people with diabetes (*n* = 3); application time for effective cauterisation (*n* = 3); potential risks associated with use of sodium hydroxide (*n* = 2); technique to minimise damage to surrounding tissues (*n* = 1)			

Trichloroacetic acid (TCA)			
Concentration	*n*	Application time	*n*
100%	9	1 min	4
90%	2	30 sec–1 min	2
80%	1	3 min	1
		10 sec	1
		2 min	1
		Various (blanching of tissues)	1
Clinical rationale for the use of TCA			5
Matrix destruction (*n* = 3); advantages when compared with phenol (*n* = 2)			
Procedural considerations for TCA			2
Limitations of TCA use for matrixectomy (*n* = 2)			

Bichloroacetic acid (BCA)			
Concentration	*n*	Application time	*n*
90%	3	4 min	2

Silver nitrate (AgNO_3_)			
Silver nitrate	1	Application	1
Silver nitrate	1	Insert stick 3–4 mm	1
Clinical rationale for the use of silver nitrate			1
Advantages over other matrixectomy methods (*n* = 1)			

Part B: Physical ablative methods			
Method	*n*	Application time	*n*
Electrosurgery			
Electrosurgery	7	Various modes	5
Clinical rationale for electrosurgery use			2
Advantages over other matrixectomy methods (*n* = 2)			
Procedural considerations for electrosurgery use			1
Risk associated with electrosurgery use (*n* = 1)			
Carbon dioxide (CO_2_) laser			
Carbon dioxide (CO_2_) laser	4	Various modes	3
Clinical rationale for carbon dioxide (CO_2_) laser			1
Enables complete nail plate matrix removal (*n* = 1)			
Radiofrequency			
Radiofrequency	3	15 sec	1
		3–5 sec rep. 2–3x	1
Clinical rationale for radiofrequency use			2
Advantages over other matrixectomy methods (*n* = 1); greater accuracy of tissue necrosis (*n* = 1)			
Procedural considerations for radiofrequency use			1
Depth of tissue damage is imprecise (*n* = 1)			
Liquid nitrogen/cryotherapy			
Liquid nitrogen/cryotherapy	2	20 sec	1
		40 sec	1
Bipolar diathermy			
Bipolar diathermy	1		
Clinical rationale for bipolar diathermy use			1
Advantages over other matrixectomy methods (*n* = 1)			
Excision without resection or incision			
Excision without resection or incision			1

*Note: n*: identifies the number of times a specific application time, rationale or a procedure consideration is discussed for the matrixectomy or irrigation methods during a non‐incisional nail surgery. A single article may present multiple techniques, application times and rationale.

Abbreviations: min = minute, *n* = number, PNA = partial nail avulsion, rep = repeat, sec = second, TNA = total nail avulsion, *X* = times.

**TABLE 3:** Summary and frequency table for post‐operative irrigation techniques and clinical rationale.

Post‐operative irrigation	Frequency (*n*)
Phenol	
Alcohol‐based solutions	41
Saline	7
Ethanol then saline	2
Ferric chloride	2
Lidocaine	1
Clinical rationale for post‐operative irrigation following phenol application	40
Neutralise phenol (*n* = 17); dilute phenol (*n* = 15); reduce caustic effect to tissue (*n* = 2); reduced oozing/bleeding (ferric chloride) (*n* = 2); not necessary as phenol is self‐limiting (*n* = 2); choice of flush to reduce toxicity to cells (*n* = 2)	
Sodium hydroxide (NaOH)	
Acetic acid 10%	7
Acetic acid 5%	4
Acetic acid 3%	1
Alcohol	1
Saline	1
Clinical rationale for post‐operative irrigation following NaOH application	11
Neutralise NaOH (*n* = 11)	
Trichloroacetic acid (TCA)	
Saline	2
Clinical rationale for post‐operative irrigation following TCA application	2
Neutralise TCA (*n* = 1); prevent excessive tissue damage (*n* = 1)	
Bichloroacetic acid (BCA)	
Saline	2
Clinical rationale for post‐operative irrigation following BCA application	1
Neutralise BCA (*n* = 1)	
Electrosurgery	
Hydrogen peroxide	1
Clinical rationale for post‐operative irrigation following electrosurgery	1
Haemostasis (hydrogen peroxide) (*n* = 1)	

*Note:* Frequency *n*: identifies the number of articles from the original sample (*n* = 110) that describe a procedural step (product, instrument, item) that is performed during a non‐incisional nail surgery. A single article may be counted in multiple categories if it addresses more than one product, instrument or item. Clinical rationale n: identifies the number of times a supporting rationale is presented for a procedural step (product, instrument, item) that is performed during a non‐incisional nail surgery. A single article may present multiple rationales.

Abbreviation: *N* = number.

### 3.2 Characteristics of Included Studies

Following full text‐screening, a total of 110 articles met the inclusion criteria for this review (Figure 1).

**FIGURE 2:**
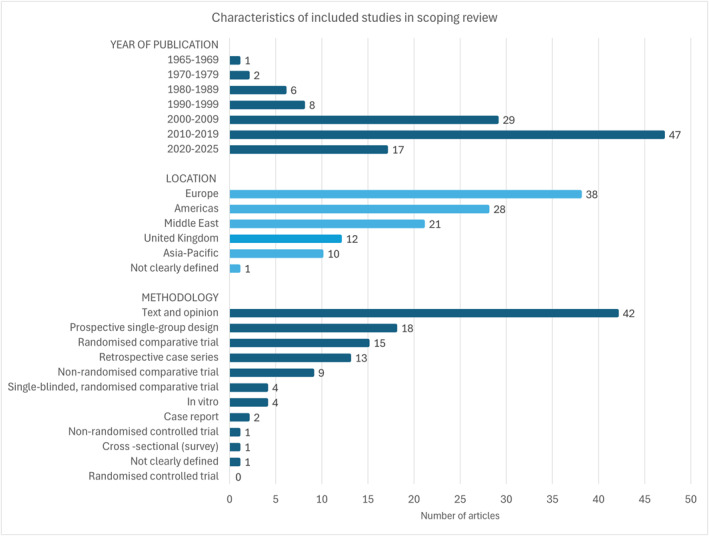
Characteristics of included articles. Year of publication of articles, location and methodology used.

Included studies were published between 1965 and 2025, with the majority originating from Europe (Figure 2). Most studies were classified as ‘text and opinion pieces’ (*n* = 42), followed by prospective single‐group designs (*n* = 18) and randomised comparative trials (*n* = 15) (Figure 2).

### 3.3 Toe Preparation

Fifty‐six studies (50.9%) reported the pre‐operative use of an antiseptic agent for toe preparation, with the most common agent being povidone‐iodine solution (*n* = 29). Other pre‐operative preparations cited by authors included a choice of either chlorhexidine, alcohol or iodine (*n* = 7), or chlorhexidine alone (*n* = 6). Of the 56 studies that described a process for toe preparation, only 10 (17.9%) provided a rationale. The most frequently cited reasons for toe preparation were to disinfect or clean the toe prior to nail surgery (Table 1, Part A).

### 3.4 Tourniquet Application

Forty‐six articles (41.8%) reported the use of a tourniquet during the surgical procedure. The use of a rubber band or latex strip was the most common option for exsanguination of the toe (*n* = 28), followed by tourniquets which were variously described as ‘sterile’, ‘digital’, ‘Esmarch’, ‘coloured ring’ or ‘mini‐tourniquets’ (*n* = 16). The primary rationale for tourniquet use was to achieve haemostasis at the surgical site and to exsanguinate the digit (*n* = 46) (Table 1, Part B).

### 3.5 Nail Plate Elevation

Use of a nail lifter was described in 45 studies (40.9%), and the most commonly reported instrument was a nail elevator (*n* = 33). The rationale offered by the majority of authors (*n* = 38) was to release the nail plate from the nail bed and surrounding soft tissue structures (Table 1, Part C).

### 3.6 Nail Plate Section

The process of sectioning the visible nail plate for a partial nail avulsion was identified in 48 (43.6%) of the studies. The most commonly used instrument was a nail splitter (*n* = 25), followed by nail nippers, scissors or a ‘side cutter’ (*n* = 19). The primary rationale for using a nail splitter was to section the toenail from the free edge to the eponychium by creating a longitudinal split in the nail (Table 1, Part D).

### 3.7 Use of a Beaver Blade

The use of a beaver blade to section the nail plate deep to the proximal nail fold was reported in only 9% of studies (*n* = 10) (Table 1, Part E).

### 3.8 Process for Nail Removal

The process for removing the nail section, or the entire nail plate, was described in 57 studies (51.8%) and was achieved using instruments such as forceps, artery clips, haemostats, and clamps. The most frequently cited rationale for this step of the nail surgery procedure was to avulse the germinal matrix or matrix horn (*n* = 10). The second most common reason was to minimise the risk of leaving residual nail fragments or spicules (*n* = 6) (Table 1, Part F).

### 3.9 Removal of Additional Tissue

Scalpels and curettes (*n* = 38) were typically used for the removal of granulation tissue, hypertrophied tissue, or granulomas (*n* = 39). This surgical process was described in 47 of the 110 studies (42.7%) (Table 1, Part G).

### 3.10 Matrixectomy Technique

Within the 110 articles reviewed, 136 descriptions of chemical matrixectomy or physical ablative techniques were identified. This reflects the choice by some authors to describe the use of multiple chemical agents, or varying concentrations of the same cauterant.

Phenol was the most commonly cited cauterant for matrixectomy (*n* = 81); 88% was the most frequently cited concentration. The majority of authors used a 3‐min application time (*n* = 31), while a 1‐min application was the second most common option (*n* = 17). The most prevalent rationale for the use of phenol was the proposed advantage of its chemical properties (*n* = 14) including: high efficacy in segmental matrix cauterisation; low recurrence rate; antimicrobial and anaesthetic properties, which contribute respectively to a reduced risk of post‐operative infection and minimal post‐operative pain. Some authors did note additional advantages of phenol for a chemical matrixectomy such as ease of application, short procedure duration, brief post‐operative recovery period, avoidance of scarring, and low recurrence rate (Table 2). In addition to the benefits offered by phenol, authors highlight the importance of balancing phenol application time with the risk of tissue destruction (*n* = 14).

The use of sodium hydroxide for chemical matrixectomy was discussed in 21 of the papers reviewed. Most of these articles (*n* = 20) described the use of a 10% solution and a 1‐min application time (*n* = 11). The advantages of the use of sodium hydroxide when compared with phenol was justified in seven of the 21 papers for reasons relating to high success rate for matrix ablation, reduced post‐operative pain, shorter duration of post‐operative drainage, and faster healing times (Table 2).

A range of other chemicals for matrixectomy was identified including trichloroacetic acid (TCA) (*n* = 12), bichloroacetic acid (BCA) (*n* = 3) and silver nitrate (*n* = 1).

Physical ablative techniques are reported less commonly in the literature including: electrosurgery (*n* = 7); carbon dioxide laser (*n* = 4); radiofrequency (*n* = 3); liquid nitrogen (*n* = 2); bipolar diathermy (*n* = 1); and excision of nail matrix without resection or incision of the proximal nail fold (*n* = 1) (Table 2). The rationale for using a physical ablative technique rather than a chemical matrixectomy focussed on the advantages of these procedures over chemical matrixectomy (*n* = 4).

### 3.11 Use of Irrigation Post‐Operatively

Post‐operative irrigation of the surgical site was described in 72 studies (65.5%). The primary rationale for irrigation was to neutralise (*n* = 17) or dilute (*n* = 15) phenol. Most authors reported the use of alcohol‐based solutions such as isopropyl alcohol, polyhexanide, methylated spirits, surgical spirit or chlorhexidine (Table 3).

Following a sodium hydroxide matrixectomy, irrigation typically involves 10% or 5% acetic acid to neutralise the sodium hydroxide (Table 3).

## 4 Discussion

To our knowledge, this is the first scoping review to explore the range of procedural steps employed in non‐incisional nail surgery, with or without matrixectomy, and the clinical rationale which informs practice. Despite the abundance of published material relating to non‐incisional nail surgery, this review identified considerable variation in surgical practice. Literature on this topic, published over a period of 37 years, is diverse in quality and methodological design with a large representation of opinion and text papers. This finding is consistent with recent systematic reviews and meta‐analyses which report inconsistent practice and highlight a generally poor quality of research in this area [7, 11].

The use of a tourniquet to achieve a bloodless field might be considered standard practice in nail surgery but this was described in less than half of the articles reviewed. This finding is inconsistent with a recent survey of UK podiatrists which reported 99.3% of 244 respondents used a tourniquet [3]. It remains unclear whether tourniquets are used regularly for nail surgery, or their use is taken for granted and not reported. The primary rationale for tourniquet use was haemostasis at the surgical site. Furthermore, some authors stated that haemostasis facilitates optimal activity of the chemical cauterant [12, 13, 14], while others suggest that a tourniquet is important as blood may neutralise, inactivate [15, 16, 17, 18, 19, 20] or counteract [21, 22] the effect of phenol. A less common perspective is that a tourniquet is utilised to prevent blood diluting the phenol, rather than neutralising its effect [23, 24, 25].

There were several papers highlighting the potential risks associated with tourniquet use, including ischemic complications due to tissue compression [20, 21, 26, 27, 28, 29]. Some authors recommended limiting tourniquet application time to 20–25 minutes [26], 20–30 minutes [30] or only applying a tourniquet immediately before the application of the caustic agent [31]. Other authors recommended caution when using a tourniquet on patients with diabetes or peripheral vascular disease [26, 27], although the nature of these precautions is not clearly defined. In contrast, some authors justified no tourniquet due to either the inclusion of adrenaline in the local anaesthetic, or the short duration of the nail removal procedure [21, 29, 32]. Overall, findings from this review suggest the rationale for tourniquet use is varied and context‐dependent, requiring careful consideration of application time and patient‐specific risk factors.

In relation to chemical matrixectomy in non‐incisional nail surgery, the use of 88% phenol is the most prevalent practice. However, the phenol concentration and application time varies considerably in the literature. Whilst a three‐minute application time is the most frequently reported protocol, the data identifies a vast range from 30 seconds to 10 minutes. A recent systematic review with meta‐analysis recommended with a moderate certainty of evidence, that a phenol application time of one‐minute was associated with a lower risk of nail regrowth, when compared to a 30 second application. The same authors also proposed that there was no additional benefit when phenol was applied for 2 or 3 minutes [7]. Recommendations to adopt a one‐minute application time are not consistent with the findings from this scoping review.

Some authors did not suggest a preferred duration for phenol application [17, 18, 31, 33, 34, 35, 36, 37, 38, 39], while others relied on observing changes in tissue colour from pink to white [40] or to a blue‐ish hue [41], adding further variability to the process of phenol application. This variability in cauterisation practice may suggest that clinical decision‐making is influenced by differing priorities. For example, a shorter phenol application time is thought to reduce tissue damage and post‐operative complications but may increase the risk of recurrence in nail re‐growth [4, 42]. Conversely, extended durations of phenol application may enhance matrix destruction but risk delayed healing and collateral tissue damage [4, 15, 23, 25, 31, 33, 34, 42, 43, 44, 45, 46, 47, 48].

Sodium hydroxide is less popular than phenol as a cauterising agent, however, authors report that it offers several advantages over phenol for matrixectomy. These benefits include: high success rates; less tissue damage, including post‐operative pain and drainage, which may lead to faster healing times [17, 26, 30, 37, 42, 45, 49]. Sodium hydroxide is reported to cause a liquefaction necrosis, resulting in more predictable tissue damage when compared with coagulative necrosis created by the application of phenol [26, 49]. The process by which tissue damage is caused may explain why some authors prefer to use sodium hydroxide for patients with poor healing, or in people with diabetes [42, 50].

This scoping review highlights significant variation in the concentration and application time of agents used for a chemical matrixectomy. There is also inconsistency in the supporting rationale and it is unclear if clinical decision‐making is informed primarily by patient specific factors, a desire to enhance post‐operative healing, or the priority of reducing the probability of nail regrowth.

The primary rationale for using physical matrix ablation over chemical matrixectomy methods focussed on reduced post‐operative pain and drainage. Physical matrixectomy techniques are reportedly quick and easy to perform [20, 28, 51] and enable greater precision in targeting matrix tissue [17, 52]. A contrasting perspective suggests that the depth and penetration of electrosurgery and radiofrequency is imprecise and the risk of collateral tissue damage is similar to that of a chemical matrixectomy [23].

The practice of irrigating a nail surgery site post‐operatively was reported in 66.4% of the studies reviewed. Typically, alcohol‐based solutions such as 70% isopropyl alcohol are used to neutralise [19, 22, 26, 27, 37, 41, 43, 53, 54, 55, 56, 57, 58, 59, 60, 61, 62] or dilute the caustic agent [4, 17, 31, 34, 38, 46, 63, 64, 65, 66, 67, 68, 69, 70, 71]. The intention of this procedural step is to prevent excessive damage to surrounding skin, reduce toxicity to periungual tissue, and to promote the healing process [36, 52]. The use of ferric chloride or hydrogen peroxide for irrigation was reported in only three studies [17, 72, 73], with the intended use relating to haemostasis and reduced post‐operative bleeding.

Some authors did provide reasons for not irrigating a nail surgery wound post‐operatively. For example, the self‐limiting action of phenol [74] and the neutralisation of phenol by blood [33]. A similar variation in practice was observed within a survey of UK podiatrists, finding that 33.2% out of 244 respondents did not irrigate [3]. Interestingly, there is an even division amongst authors who report that post‐surgical irrigation is an important step to neutralise phenol (*n* = 17), as opposed to diluting phenol (*n* = 15). Standardising irrigation methods, based on the chemical agent applied, may improve wound healing times and reduce the risk of post‐operative infection.

### 4.1 Summary & Recommendations

Non‐incisional nail surgeries are commonly performed by clinicians to manage infected and recurrent IGTN. However, the selection of surgical procedure or choice of technique is highly varied, and the clinical rationale informing clinical decision‐making is often unclear or not reported. By systematically mapping the literature, this review provides a comprehensive overview of how knowledge in non‐incisional nail surgery has been reported. Although not intended to assess effectiveness, it does establish a clear evidence landscape, identifies dominant sources and gaps, and offers a valuable foundation to guide future research.

This review highlights the need for a deeper understanding of decision‐making and clinical rationale which informs non‐incisional nail surgery practice. Rationales identified within the literature draw on a mix of opinion‐based, narrative, and primary sources. Therefore, inconsistencies in clinical decision‐making may reflect a reliance on historic practice, underscoring the need for contemporary, robust research to inform clear clinical guidance.

A major challenge for future research and guideline development lies in the heterogeneity of the existing literature, particularly in terms of methodological approaches and the quality of evidence. Further studies are required to improve understanding about the influence of patient‐specific factors such as medical history and presenting pathology, aiming to standardise procedural approaches to non‐incisional nail surgery with or without matrixectomy. Key areas where future comparative research would be most valuable may include the effect of blood on phenol, the optimal time and concentration of phenol application for healing and prevention of regrowth, the benefits of sodium hydroxide matrixectomy compared with other techniques, and the impact of different irrigation methods on postoperative healing.

The findings of this review should be interpreted in the context of several limitations within the available literature. No systematic search can capture all potentially relevant evidence, and it is possible that papers discussing technique and rationale in relation to non‐incisional nail surgeries were missed with the search terms and inclusion criteria applied. It is also possible that some authors may have not reported aspects of the surgical process, or their clinical rationale. The authors propose that this review has highlighted the variability of practice in non‐incisional nail surgery and identified a need for clinical guidelines to provide a structured decision‐making framework and promote consistency of practice.

## Author Contributions

**Anna Horn:** conceptualization, data curation, formal analysis (lead), project administration, writing – original draft, writing – review and editing. **Caroline Robinson:** conceptualization, data curation, formal analysis (support), writing – review and editing. **Luke Donnan:** conceptualization, data curation, formal analysis (support), writing – review and editing.

## Funding

The authors have nothing to report.

## Conflicts of Interest

The authors declare no conflicts of interest.

## Supporting information


Supporting Information S1

Supporting Information S2

Supporting Information S3

## Data Availability

The data that supports the findings of this study are available in the supplementary material of this article.
