# Supplementary material for: Clinical Rationale for Procedural Steps When Performing Non‐Incisional Nail Surgery: A Scoping Review
Source: J Foot Ankle Res. 2026 Jul 27;19(3):e70191. doi: 10.1002/jfa2.70191 (PMC13408232; doi:10.1002/jfa2.70191)
Supplement: Supplementary file 1 — Supporting Information S1 [file JFA2-19-e70191-s001.docx]

**Clinical rationale for procedural steps when performing non-incisional nail surgery: a scoping review**

**Appendix A: Database search strategy**

ingrow* OR onychocryptosis OR involuted OR embedded OR pincer

**AND**

surger* OR matrixectomy OR matricectomy OR curettage OR phenol* OR ablation OR avulsion OR electrosurg*

**AND**

nail* OR toenail
